# Which pediatric practices use substance use consultation services?

**DOI:** 10.3389/fped.2024.1337944

**Published:** 2024-07-16

**Authors:** Sharon Levy, Mei-Sing Ong, Machiko Minegishi, Melissa Brogna, Shannon Mountain-Ray, Elyse Neubauer, Jonas Bromberg, John Straus, Elissa R. Weitzman

**Affiliations:** ^1^ Division of Addiction Medicine, Boston Children’s Hospital, Boston, MA, United States; ^2^ Department of Pediatrics, Harvard Medical School, Boston, MA, United States; ^3^ Department of Population Medicine, Harvard Medical School & Harvard Pilgrim Health Care Institute, Boston, MA, United States; ^4^ Department of Psychiatry and Behavioral Sciences, Boston Children’s Hospital, Boston, MA, United States; ^5^ Behavioral Health Integration Program, Pediatric Physicians’ Organization at Children’s, Boston Children’s Hospital, Boston, MA, United States; ^6^ Massachusetts Child Psychiatry Access Program, Beacon Health Options, Anthem, Boston, MA, United States; ^7^ Division of Adolescent and Young Adult Medicine, Boston Children’s Hospital, Boston, MA, United States; ^8^ Computational Health Informatics Program, Boston Children’s Hospital, Boston, MA, United States

**Keywords:** adolescents, substance use, treatment, primary care, alcohol, tobacco, cannabis

## Abstract

Substance use disorders (SUD) are common in adolescents and young adults, though few youth with SUD receive treatment, and rates of medication for substance use disorder treatment are much lower in youth compared to adults. Pediatric primary care could present an opportunity for youth with SUD to access medication, though pediatric providers may need support. Massachusetts has provided a substance use consultation line for pediatric providers since 2018. One large network of independent primary care practices within the state has been further supported by access to resources provided through a grant from the Substance Abuse and Mental Health Services Administration. In this paper, we describe the services provided in Massachusetts and examine whether additional resources are associated with increased use of the consultation line as a marker of provider engagement in SUD treatment.

## Introduction

According to the National Survey on Drug Use and Health, 34.0% of youths aged 12–17 and 49.1% aged 18–25 reported that a health professional inquired about their alcohol use in the past year (1). Similarly, 31.7% of those aged 12–17 and 44.2% of those aged 18–25 reported that a health professional asked about their drug use during the same period (1). In the same year, 8.5% of US adolescents aged 12–17, and 14.1% of young adults aged 18–25 met criteria for a substance use disorder in 2021 (1), yet fewer than 9% of youth who needed treatment received it (2). Given the large unmet need, expanding substance use treatment to primary care settings is a logical strategy.

The American Academy of Pediatrics (AAP) recommends that pediatricians incorporate Screening Brief Intervention Referral to Treatment, or SBIRT framework, into routine medical care to identify substance use and intervene, including preventive messaging for those with no past year use to brief interventions among those who report use. However, SBIRT does not typically include recommended medications for substance use disorders (3–5), leaving a tremendous access gap for youth. Although pediatric primary care patients are often considered a low-risk group, in a study performed during the same timeframe, we found that approximately 1%–6% of pediatric primary care patients screened for substance use met criteria for an alcohol, tobacco/nicotine, or cannabis use disorder (6). Indeed, receipt of medication treatment for opioid and nicotine use disorders is correlated with age (7–9). Creating access points for SUD medications in pediatric primary care could address this gap.

Historically, little curriculum time is devoted to Addiction Medicine training during medical school (10). Pediatric residencies are now likely to include some substance use education, though substantial gaps remain, including around medication prescribing (10). The nationwide shortage of behavioral health professionals (11) further limits access and complicates the provision of SUD treatment in pediatric primary care. Strategies to increase capacity are needed.

Our group previously demonstrated that providing pediatricians access to consultations from Addiction Medicine specialists combined with access to Behavioral Health (BH) counselors for pediatric patients was feasible and that most patients (68%) for whom consults were requested could be managed in primary care (12). We have hypothesized that augmenting support around substance use treatment for pediatric practices through activities such as educational programming, practice-wide discussions and feedback on screening and referral rates would increase use of these services. In this report we analyzed administrative data to determine whether augmented services increase use of calls to a pediatric consultation call line.

## Method

We used administrative data captured from provider-to-provider consultations and billing data from the provision of counseling appointments to analyze correlates of Addiction Medicine consultation service utilization among pediatric primary care practices in Massachusetts. This project was determined to be exempt by the Boston Children's Hospital Institutional Review Board.

### Program description

This project has been previously described in detail (12) and is summarized here.

#### Addiction medicine consultations and referrals to virtual counseling

Beginning in July 2019, the state of Massachusetts began offering pediatric substance use consultations from Addiction Medicine specialists to primary care pediatricians through an expansion of the statewide Massachusetts Child Psychiatry Access Program (MCPAP) (13). Pediatric providers practicing within the state access the service by calling their regional MCPAP phone number during normal business hours. Trained MCPAP administrators triage questions to the appropriate consultant, usually a child psychiatrist at an academic medical center. With the start of this new service, questions about substance use were forwarded to consultants who were members of the faculty of the Adolescent Substance Use and Addiction Program (ASAP) or an Addiction Medicine Fellow at Boston Children's Hospital. Primary specialties of BCH SUD consultants include General Pediatrics, Developmental-Behavioral Pediatrics, Family Medicine, Preventive Medicine and Child and Adolescent Psychiatry; consultants are board-certified or board-eligible in Addiction Medicine or a nurse practitioner with extensive experience in adolescent substance use disorders. Each consultation request is entered into a secure electronic database by an administrative assistant and then completed by the consultant after the consultation. In October 2021, the service was expanded to include referrals to virtual SUD counseling for pediatric primary care patients in addition to support from an Addiction Medicine specialist. The substance use consultation service was advertised to all practices that are registered with MCPAP by placing notices in MCPAP quarterly newsletters, e-mail announcements and via the MCPAP “clinical conversations” webinar series which is typically attended by 15–30 clinicians and posted on the MCPAP website. Consultants and BH counselors also promoted the service informally. More than 95% of pediatric primary care practices in MA are enrolled in the MCPAP (12).

#### The pediatric physicians' organization at children's (PPOC)

The PPOC is a network of independent community-based pediatric primary care practices affiliated with Boston Children's Hospital. The PPOC network emphasizes and supports pediatricians' efforts to address behavioral health problems, including substance use, through a Behavioral Health Learning Community (BHLC). The BHLC is a two-year course designed to increase the pediatric primary care team's knowledge, skills, and confidence in identifying, assessing, and treating mild to moderate presentations of common child and adolescent psychiatric and behavioral health disorders, including substance use disorders. For each group of diagnostic conditions, the BHLC curriculum covers the etiology, identification, course, prevention, and use of evidence-based treatments. The course places special emphasis on developing primary care physician's ability to use standardized screening tools to identify behavioral health and substance use concerns, to establish baseline symptom severity, and measure response to treatment. The course includes five hours of substance use content (alcohol, cannabis, opioids, vaping, and drug testing) taught by an ASAP faculty member.

Starting in 2018, the Addiction Medicine program at Boston Children's Hospital partnered with the PPOC to deliver a tailored model of substance use service to a subset of practices which increased over time. The effort, which was supported by a federal grant from the Substance Abuse and Mental Health Services Administration (SAMHSA, TI081137), provided partner practices with additional substance use training resources and access to a specially trained behavioral health counselor to work with pediatric primary care patients. The program, which initially embedded a specially trained behavioral health counselor to deliver in person counseling to pediatric primary care patients with substance use problems, was redesigned and converted into a virtual model in 2020 due to the COVID-19 pandemic. With the widespread acceptance of virtual care after the pandemic, the program centralized behavioral health counselors to maximize efficiency by allowing multiple practices to refer to a single BH specialist. Over time, training content was developed to reflect feedback from primary care providers. Printed and recorded materials developed for this project were made available to the entire PPOC network. Table 1 describes the substance use supports available to PPOC practices.

**Table 1:** Description of substance use learning content and services provided to PPOC practices, 2019-2023.

Services available to entire MCPAP network	No. Practices
MCPAP Pediatric primary care providers can access to telephone or Face-to-face consultation with either a Child and Adolescent Psychiatrist or independently licensed Behavioral Health Clinician	^a^
Referral to a team behavioral health clinician for a diagnostic or acute face-to-face assessment	
Recommendation for the family to access local behavioral health services with support from either the practice's care coordinator or behavioral health clinician or the MCPAP team's resource and referral specialist	
Telehealth counseling by ASAP-MCPAP's substance use counselor for adolescents who use substances if assessed as appropriate.	
MCPAP Pediatric primary care providers have access to education and training relative to psychiatric disorders and medications to improve integration of behavioral health with primary care.	
Services available to entire PPOC network	No. Practices
Behavioural Health Learning Community: 2-year course for primary care physicians covering, 27 h of content including 5 h devoted exclusively to substance use	90+
Substance use screen and clinical guidance built into electronic medical record	
Access to recorded core trainings and referral instructions via online portal	
6-session buprenorphine waiver/prescribing training	
Access to 27 “Tip Sheets” for managing adolescent substance use problems	
Additional SAMHSA funded service^b^	30
Access to embedded or centralized BH counsellors for evaluation, ongoing treatment, parent guidance and referral support	
Clinical progress reports for each patient in BH counselling	
Individual practice level trainings on pediatric substance used^c^	
Quarterly reports of screening rates and summative screen results	
Routine, recurrent meetings with Addiction Medicine and BH counselling staff	

PPOC, The Pediatric Physicians’ Organization at Boston Children's Hospital; SAMHSA, Substance Abuse and Mental Health Services Administration; BH, Behavioural Health.

^a^ Total MCPAP enrolled practices. Approximately 95% of all the pediatric primary care practices in MA have been enrolled in the program.

^b^ Steadily increased from 3 in Year 1.

^c^ Training include the following: Introduction to adolescent substance use, screening and brief intervention, “handoff” to BH counselling, alcohol/naltrexone, nicotine and cannabis, stigma, behavioral contracts, urine drug testing, the impact of disruptions and COVID, MI and Brief Intervention for adolescents.

#### Consultations initiated by addiction medicine consultants

To optimize operational efficiency and reduce wait times for new patients requesting a substance use disorders evaluation in the ASAP subspecialty program, beginning in 2021, Addiction Medicine consultants *initiated* some consultations with primary care providers for patients on waitlist who were assessed by clinical intake form to be appropriate for management in primary care. The consultation service was available for all the practices enrolled in the MCPAP program. Adolescents who were willing to participate in virtual counseling were offered the opportunity to take advantage of this program. In these cases, a staff member discussed with patient and/or parent the option of receiving substance use treatment with their primary care provider and a BH counselor through the Division of Addiction Medicine; if interested, consent to release information was obtained and a consultant called the primary care provider to discuss the case. If the primary care provider was willing to manage SUD treatment in conjunction with a BH counselor and with the support of Addiction Medicine consultants, the patient was eligible for this service. The Addiction Medicine team considered patients who were judged to be at high risk of overdose based on a presenting complaint or primary care provider report of opioid use, sedative use or heavy alcohol use, adolescents who reported they did not have access to a confidential space for counseling sessions, those who were considered by their primary care provider to be unable to participate in virtual visits due to developmental or cognitive problems and those for whom there were concerns of domestic or interpersonal violence or child maltreatment ineligible for virtual counseling and instead were seen in the ASAP subspecialty program. Conversations with primary care providers were captured as “consultations” even though they were initiated by the Addiction Medicine team. This practice continued throughout the period of this study, though the practice decreased significantly after 2022 as virtual BH counselors' caseloads grew, and the program had less capacity to accommodate new patients. The precise number was tracked from March 2022 through the end of our study period: a total of 71 consultations were initiated by Addiction Medicine consultants from March-December 2022 and 10 from January through October 2023. Referrals for adolescents whose primary care provider was part of the PPOC network went through a separate pathway that did not involve Addiction Medicine consultants initiating consultations.

### Sources of data

#### MCPAP electronic database

Each consultation request was entered into a secure electronic database that is compliant with the Health Insurance Portability and Accountability Act (HIPAA) of 1986. The encounter data fields include patient demographic information (age, sex, insurance plan, and de-identified member number), primary care practice, provider and encounter type (which were entered by an administrative assistant) and substance use concern, medication and outcome recommendations (entered by the consultant). All identifiable patient information is encrypted and available only to consultants. The database is hosted by MCPAP, a third-party contractor to the Massachusetts Department of Mental Health. Data summaries were provided by one of the authors (JS) who is the Founding Director of MCPAP. The data regarding the exact size of the participating practices was not readily accessible. No personal health information was included in the database summary.

### Data analysis

Administrative data for consultation requests were collected between the First Quarter of 2020 (1 October 2019–31 December 2019) and the Fourth Quarter of 2023 (1 July 2023–30 September 2023). Practices were considered part of the PPOC network if they were included in the PPOC at any point during the study period.

The primary outcome was trends in the utilization of Addiction Medicine consultation service over time among PPOC practices, compared against non-PPOC practices. Specifically, we quantified the proportion of practices utilizing the Addiction Medicine consultation service among PPOC practices, and separately among non-PPOC practices, in each year of the study period. We then examined if the trends in utilization varied across the study period for these 2 groups of practices.

Cochran's Q test was used to evaluate whether the overall trend varied across the study period for PPOC and non-PPOC practices separately. Pairwise McNemar Tests were used to assess whether there were significant differences in the proportion of practices that utilized the Addiction Medicine consultation service between adjacent years within each group.

The overall number of consultation requests from all MCPAP program practices (both PPOC and non-PPOC practices) reported quarterly was visually assessed using a loess (locally weighted scatterplot smoothing) method applied to examine the potential trend in the data. The smoothing parameter was set to 0.5 to balance the trade-off between retaining local variation and achieving overall smoothness in the trend line.

The SAS statistical software (9.4) and R 4.3.2 (R Core Team, 2023) were used in all statistical analyses. Two-sided *p*-values < .05 were considered statistically significant.

## Results

There were 139 participating practices in the MCPAP program during the study period, each of which used the Addiction Medicine consultation service at least once. Among these, one practice that joined the MCPAP program in 2022 and eight practices with uncertain PPOC status were excluded, leaving a total of 130 practices in the analytic dataset.

Of the 130 practices included in our study, 50 (38.5%) were members of the PPOC network. A total of 438 Addiction Medicine consultation requests were made by these practices between 2020 and 2023 (Table 2). The total number of consultation requests increased from 49 in 2020 to 191 in 2022, and fell to 94 in 2023 (Supplementary Figure S1). There was also heterogeneity in the use of consultation calls among practices (Supplementary Figure S2).

**Table 2:** Utilization of MCPAP program addiction medicine consultation calls, 2020–2023 (*N* = 130 practices).

Year (s)	Non-PPOC, *N* = 80 (Practices)				PPOC, *N* = 50 (Practices)			
	Consultation Calls^a^, total	Practices Using Consultation, *n* (%)^b^	McNemar test *P*-value^c^	Cochran's Q test *P*-value^d^	Consultation Calls^a^, total	Practices Using Consultation, *n* (%)^b^	McNemar test *P*-value^c^	Cochran's Q test *P*-value^d^
2020	15	13 (16.2%)	NA	<0.001	34	18 (36.0%)	NA	0.14
2021	60	35 (43.8%)	<0.001		44	23 (46.0%)	0.38	
2022	117	60 (75.0%)	<0.001		74	30 (60.0%)	0.25	
2023	39	25 (31.2%)	<0.001		55	24 (48.0%)	0.29	

MCPAP, Massachusetts Child Psychiatry Access Program; PPOC,The Pediatric Physicians’ Organization at Boston Children's Hospital.

^a^ The total annual number of Addiction Medicine consultation calls from the practices.

^b^ The percentage of practices within the group that utilized Addiction Medicine consultation calls.

^c^ The McNemar test was used to compare the annual changes in the proportion of Addiction Medicine call utilization within each group for consecutive years: 2020–2021, 2021–2022, and 2022–2023.

^d^ Cochran's Q test was used to evaluate the consistency in the proportion of Addiction Medicine call utilization within each group over the period 2020–2023.

**Figure 1:**
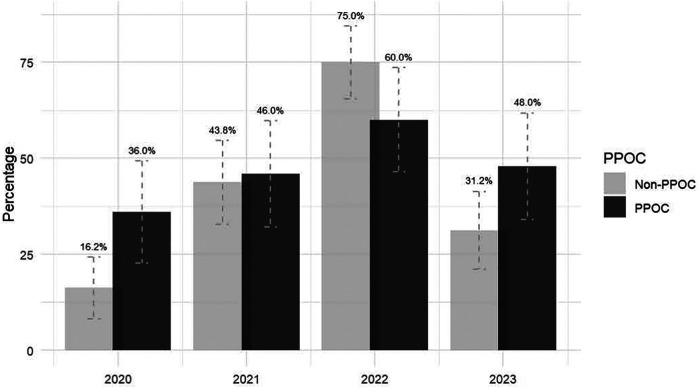
Utilization trends of MCPAP program addiction medicine consultation service across practices: 2020-2023.

Among PPOC practices, the proportion of practices utilizing the Addiction Service consultation service did not vary significantly throughout the observation period, from 36.0% in 2020 to 48% in 2023 (*P* = .14), However, among non-PPOC practices, consultation service utilization varied significantly across the study period. Notably, while the proportion of non-PPOC practices utilizing the consultation service increased from 2020 to 2021 (16.2%–43.8%; *P* < .001) and from 2021 to 2022 (43.8% to 75.0%; *P* < .001), it decreased substantially in year 2023 (75.0%–31.2%; *P* < .001) (Figure 1).

## Discussion

In this paper we describe a series of services that were provided by Addiction Medicine specialists and specially trained behavioral health counselors to provide various levels of support for substance use treatment in pediatric primary care in the state of Massachusetts. All practices within the state had access to a phone consultation line and behavioral health referrals; the full range of services included teaching/training sessions, quality improvement activities, written tip sheets and guidelines, and embedded or regional counselors specially trained in supporting adolescents with substance use disorders and their families. In this analysis, we considered use of Addiction Medicine consultations a marker of providing substance use treatment for pediatric primary care patients.

We found that consultation requests to a Pediatric Addiction Medicine call line increased over the first few years, in association with advertising. At peak volume during this period, the service fielded 191 calls, with nearly 70% of MCPAP program practices utilizing the Addiction Medicine call lines. It is our assumption that primary care providers accustomed to treating substance use would not need the support of a consultant for every patient, as is the case for other conditions that are treated in primary care, though we also assume that primary care providers that routinely address substance use will have more questions for consultants.

Primary care practices that participated in the PPOC network that emphasized SUD treatment in pediatric primary care and had access to more services used the consultation service more actively: while the rate of consultations received from practices in this network was similar to outside practices, all of the consultations were initiated by primary care providers. The rate of consultations from within the network was sustained over time, while the rate of consultations from outside practices fluctuated, in association with the number of calls initiated by consultants. Furthermore, many primary care providers within the PPOC network had access to BH counselors through a separate pathway and did not need the consultation line simply to access behavioral health counseling, resulting in fewer MCPAP consultation calls from them. Despite these, the calls from the PPOC practices were constant. Compared to calls from providers in non-PPOC practices, calls from PPOC providers were more likely to be focused on the medical management of patients with substance use disorders.

### Limitations

This project examined the utilization of a service available to all pediatric primary care providers in the Commonwealth of Massachusetts. As such, it included practices that care for youth across a spectrum of socio-economic statuses, from urban, suburban and rural areas, and from diverse racial and ethnic backgrounds. However, generalizability to other areas is not known. Although we used zip code information to quantify the number of youth in the catchment area, using only the zip code of the address of the practice may not represent the true population of the catchment area of the practice. The effort to provide augmented services within a network of primary care practices was not designed as a research effort, thus this analysis is based on administrative data, which was not captured as rigorously as it would have been in a research setting. The program evolved continuously over the course of the study period in response to both the COVID-19 pandemic and the needs of pediatric primary care providers. While our data suggests that access to a wide range of support services was associated with greater use of a consultation line, we do not know which, if any, of these services this can be attributed to. It is possible that non-PPOC practices received support from other specialists. However, we are not aware of any other organized effort to provide consultation for managing substance use in pediatric primary care within Massachusetts, and thus we believe the extent that providers sought guidance elsewhere was likely limited. The database that was used for this report does not have any patient level outcomes. A future study that examines patient outcomes among pediatric primary care patients whose providers had access to services compared to those who did not would be important in evaluating the overall impact of this practice.

We conclude that providing supports to primary care providers appears to increase attention to substance use disorder treatment in pediatric primary care. While the rates of substance use disorders among pediatric primary care patients may be relatively low, creating a multitude of access points for youth may be needed to address the very low rates of SUD treatment in this age group. Furthermore, it is possible that promoting attention to substance use treatment in primary care settings where children are followed over time may enable earlier SUD treatment, before the most serious consequences of substance use disorders accrue.

## Data Availability

The data analyzed in this study is subject to the following licenses/restrictions: The dataset used for this article reflects information about clinical encounters for current patients. Requests to access these datasets should be directed to sharon.levy@childrens.harvard.edu.
