# Supplementary figures and images for: Which pediatric practices use substance use consultation services?
Source: Front Pediatr. 2024 Jul 16;12:1337944. doi: 10.3389/fped.2024.1337944 (PMC11286468; doi:10.3389/fped.2024.1337944)

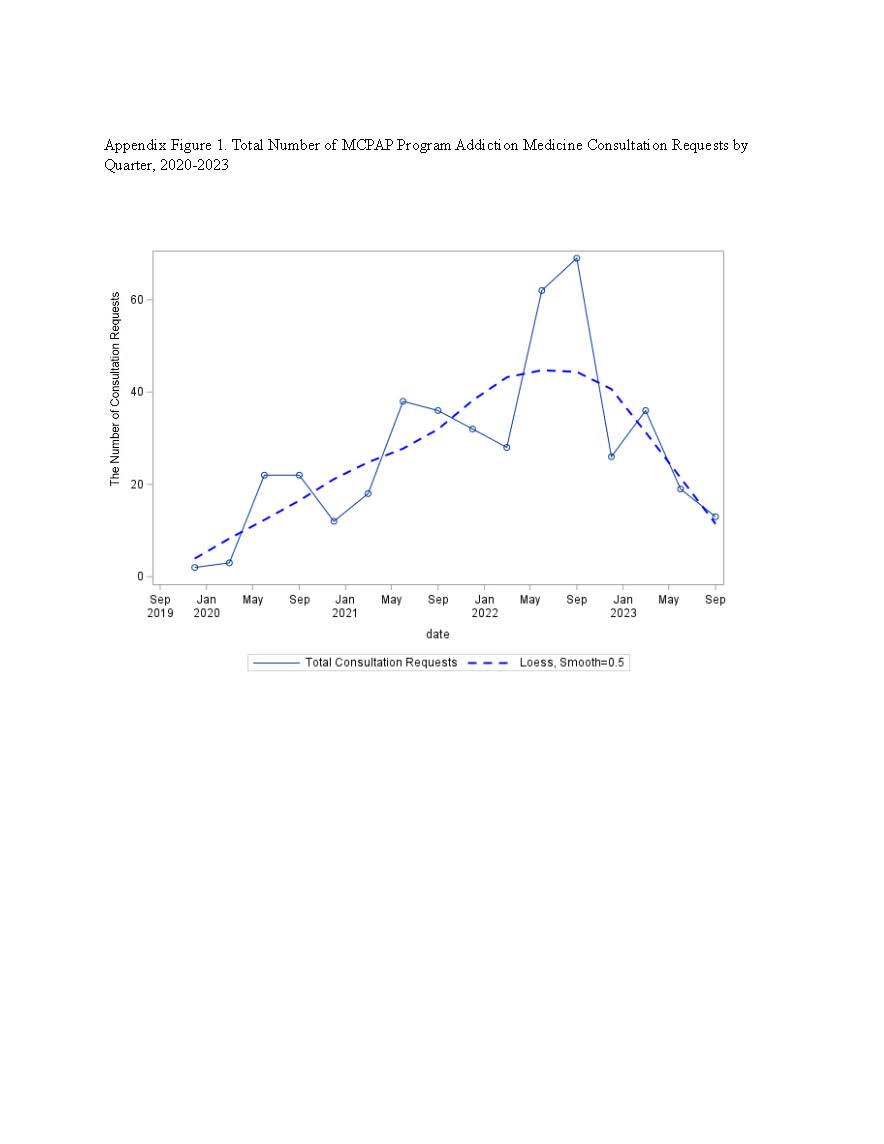

Supplement: Supplementary file 1 [file Image1.jpeg]

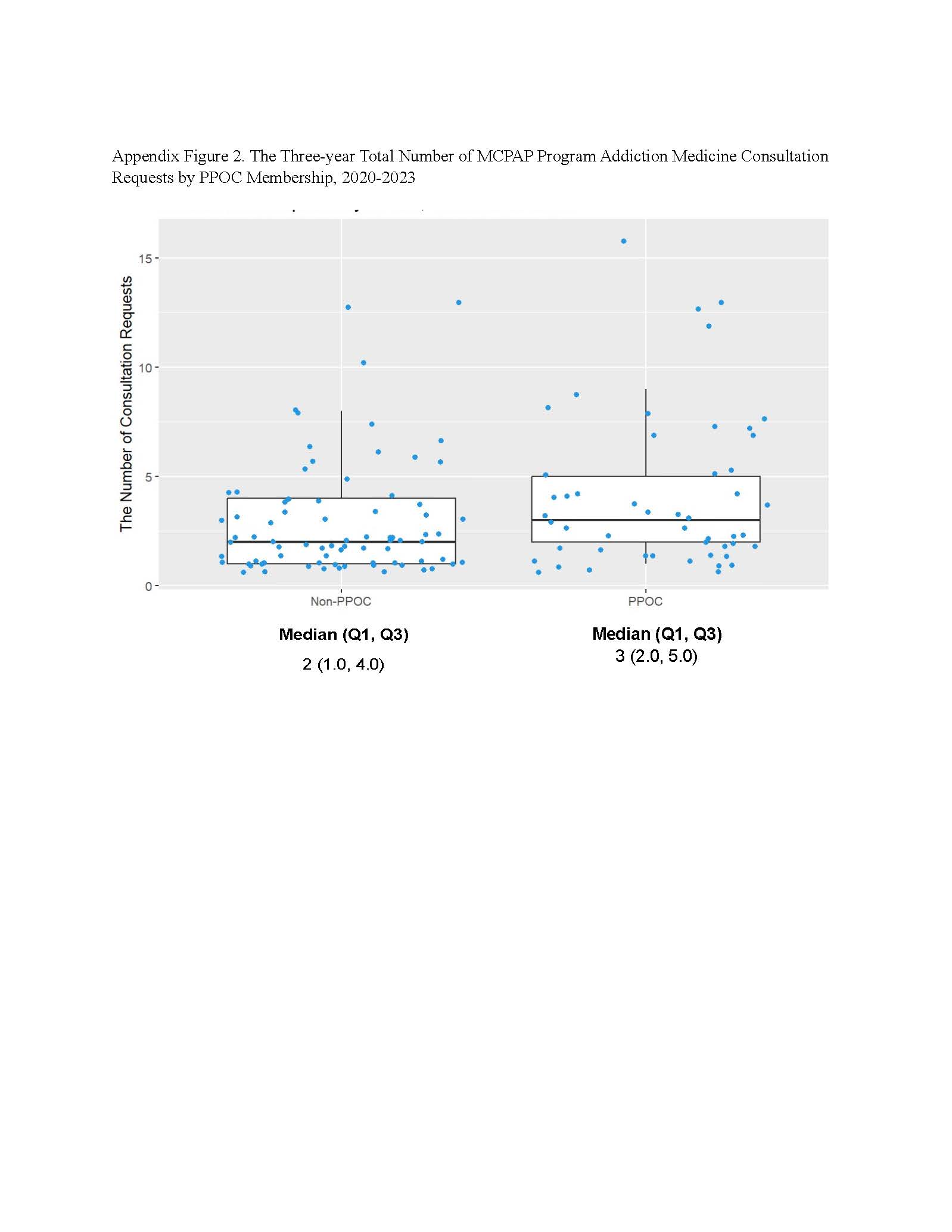

Supplement: Supplementary file 2 [file Image2.jpeg]
